# Perindopril Ameliorates Sodium Valproate-Induced Rat Model of Autism: Involvement of Sirtuin-1, JAK2/STAT3 Axis, PI3K/Akt/GSK-3β Pathway, and PPAR-Gamma Signaling

**DOI:** 10.3390/medicina60111802

**Published:** 2024-11-03

**Authors:** Anwar M. Alnakhli, Asmaa Saleh, Ahmed M. Kabel, Remon S. Estfanous, Hany M. Borg, Khulud M. Alsufyani, Nesreen M. Sabry, Fatma Alzahraa M. Gomaa, Maaly A. Abd Elmaaboud

**Affiliations:** 1 Department of Pharmaceutical Sciences, College of Pharmacy, Princess Nourah Bint Abdulrahman University, P.O. Box 84428, Riyadh 11671, Saudi Arabia; amalnklee@pnu.edu.sa (A.M.A.); asali@pnu.edu.sa (A.S.); 2 Department of Pharmacology, Faculty of Medicine, Tanta University, Tanta 31527, Egypt; 3 Anatomy and Embryology Department, Faculty of Medicine, Tanta University, Tanta 31527, Egypt; remon.astfanous@med.tanta.edu.eg; 4 Physiology Department, Faculty of Medicine, Kafrelsheikh University, Kafr El-Shaikh 33516, Egypt; 5 Taif Medical Center, Taif 26526, Saudi Arabia; 6 Clinical Oncology Department, Faculty of Medicine, Tanta University, Tanta 31527, Egypt; nesreen.afefy@med.tanta.edu.eg; 7 Pharamcognosy and Medicinal Herbs Department, Faculty of Pharmacy, Al-Baha University, AlBaha 65779, Saudi Arabia; fgomaa@bu.edu.sa

**Keywords:** autism, perindopril, valproic acid, neuroinflammation, apoptosis, rats

## Abstract

*Background and Objectives:* Autism is a developmental disability characterized by impairment of motor functions and social communication together with the development of repetitive or stereotyped behaviors. Neither the exact etiology or the curative treatment of autism are yet completely explored. The goals of this study were to evaluate the possible effects of perindopril on a rat model of autism and to elucidate the possible molecular mechanisms that may contribute to these effects. *Materials and Methods:* In a rat model of sodium valproate (VPA)-induced autism, the effect of postnatal administration of different doses of perindopril on growth and motor development, social and repetitive behaviors, sirtuin-1, oxidative stress and inflammatory markers, PI3K/Akt/GSK-3β pathway, JAK2/STAT3 axis, and PPAR-gamma signaling in the hippocampal tissues were investigated. The histopathological and electron microscopic changes elicited by administration of the different treatments were also investigated. *Results*: Perindopril dose-dependently combatted the effects of prenatal exposure to VPA on growth and maturation, motor development, and social and repetitive behaviors. In addition, the different doses of perindopril ameliorated the effects of prenatal exposure to VPA on sirtuin-1, oxidative stress and inflammatory markers, PI3K/Akt/GSK-3β pathway, JAK2/STAT3 axis, and PPAR-gamma signaling. These effects had a mitigating impact on VPA-induced histopathological and electron microscopic changes in the hippocampal tissues. *Conclusions*: Perindopril may emerge as a promising agent for amelioration of the pathologic changes of autism spectrum disorders.

## 1. Introduction

Autism is a group of developmental disability disorders that include significantly decreased social communication together with abnormality in social behavior with restriction and repetition [1]. Although its underlying pathogeneses have not been well characterized yet, genetic background, epigenetic mutations, nutritional deficiencies, and exposure to environmental pollutants during pregnancy are the mostly incriminated factors [2]. Biochemical disturbances such as abnormal redox status and mitochondrial dysfunction together with activation of the inflammatory cascade represent characteristic features of autism [3].

Recent studies reported that certain areas of the brain are affected by autism leading to significant interference with the brain functions [4]. These areas include the hippocampus, the cerebellum, and the frontal lobe [5]. Recent reports had detected certain structural and functional changes in the hippocampus more than the other areas of the brain in cases with autism which may occur as a consequence of affection of the cerebral blood flow and metabolism together with interference with the normal connections between the different areas of the brain [6]. In addition, cerebellar dysfunction may play a key role in the pathogenesis of autism [7]. Cerebellar structural and functional developmental abnormalities were reported to be clearly noticeable in patients with autism [8]. These abnormalities are associated with cognitive and motor behavioral defects and social reward processing [9].

Accumulating data suggested that Janus kinase 2 (JAK2)/signal transducer and activator of transcription-3 (STAT 3)/peroxisome proliferator-activated receptor (PPAR) gamma signaling pathway might play a pivotal role in the pathogenesis of autism [10]. Activation of JAK2 in cases of autism induces phosphorylation of STAT3 in the astrocytes and microglia which in turn elicits profound damage to the neural tissues [11]. Interestingly, PPAR-gamma prevents phosphorylation of STAT 3, thus greatly contributing to the prevention of the neurological complications of autism [12]. In addition, affection of phosphoinositide-3-kinase/Akt/glycogen synthase kinase 3 beta (PI3K/Akt/GSK-3β) signaling may be incriminated in the pathogenic events that occur in autism [13]. Cases with autism were found to have defective PI3K/Akt signaling which was proven to be involved in the survival of the neurons and their differentiation [14]. On the other hand, GSK-3β levels were significantly elevated in the brain in cases with autism and were proven to augment apoptosis and have a damaging effect on neuronal development [15].

Several animal models were utilized for investigating autism including genetic, environmentally induced, drug-induced, and idiopathic models [16]. Children with autism spectrum disease (ASD) show oxidative stress and epigenetic damage which could be related to exposure to chemicals, toxins or environmental pollutants. This exposure usually occurs via the transplacental passage of these neurotoxins during pregnancy [17]. The drug-induced ASD animal model is simple and fast with less cost, and can be used for studying central nervous system functions with the net result of finding new targets for the treatment and screening of new drugs [18]. Several studies had proven that valproic acid (VPA)-induced autism represent the most reliable model of this disorder [19]. Prenatal and postnatal exposure to valproic acid (VPA) was proven to be associated with autism-like behavior in animals and affect hippocampus-related functions such as learning and memory [20]. This model also induces oxidative stress and neuroinflammation, causes an imbalance between the excitatory and the inhibitory neurotransmitters which affects the neurotrophic factors related to neuroplasticity and neurogenesis, and alters the expression of ASD-related genes [21]. The prenatal exposure to VPA is found to be superior to postnatal exposure in inducing the behavioral and neurobiological changes [22]. The VPA-induced ASD model was also used in the current study due to its clinical relevance to human phenotype except linguistic affection and affection of serotonin levels in the brain regions as in the human studies [23]. Most drugs used in the management of autism only treat secondary symptoms of anxiety, aggression, and depression without improving the core symptoms such as social impairment [24]. Subsequently, the VPA-induced ASD model may represent an important clue to studying the underlying pathologic features of autism and to explore the effects of various suggested novel therapeutic remedies for this disorder [25].

Perindopril is a member of angiotensin-converting enzyme inhibitors that have proven efficacy in the treatment of cardiovascular disorders including hypertension, angina, and myocardial infarction [26]. Recent reports suggested that perindopril might possess potent protective effects in a wide variety of neurological diseases [27]. This might be attributed to its ameliorative effects on oxidative stress and the inflammatory cascade [28]. These effects together with its ability to modulate autophagy/apoptosis balance may represent a strong basis for the introduction of perindopril as a protective agent against experimentally induced autism [29]. The experiments performed in the present study aimed at investigating the effects of perindopril on a rat model of autism and elucidating the possible mechanisms that may contribute to these effects.

## 2. Materials and Methods

### 2.1. Ethical Considerations

All the experiments performed in the current study complied with the ARRIVE guidelines and were performed in accordance with the U.K. Animals (Scientific Procedures) Act, 1986 and associated guidelines, EU Directive 2010/63/EU for animal experiments. Furthermore, reporting of animal testing experiments complied with the ARRIVE guidelines. An approval for the protocol of this study was attained from the Research Ethics Committee of Faculty of Medicine, Tanta University, Egypt (Approval code 36264PR724/6/24; Date of approval 3 June 2024).

### 2.2. Compounds and Chemicals Used

Sodium valproate (VPA) and dimethyl sulfoxide (DMSO) were purchased in a powder form from Tokyo Chemical Industry Co., Chuo-ku, Tokyo, Japan (CAS number 1069-66-5 and 67-68-5, respectively). Perindopril was supplied in a powder form by Cayman Chemical Co., Ann Arbor, MI, USA (CAS number 82834-16-0). VPA was dissolved in normal saline. Perindopril was dissolved in 10% dimethyl sulfoxide (DMSO) solution. Sigma Aldrich Chemical Co., St. Louis, LO, USA supplied all other compounds and chemicals used in this study which were of analytical grade.

### 2.3. The Experimental Protocol

Forty adult Wistar rats of both sexes weighing 170–230 g, obtained from a local source, were utilized in this study. These animals were housed in wire mesh cages at 22 ± 4 °C with relative humidity of 52 ± 8%. Animals had free access to a rat-standard diet and water ad libitum. These animals were allowed to mate overnight and in the next morning, the presence of the vaginal plug was considered as a verification of pregnancy. This day was regarded to in the current work as gestational day 0 (GD 0). Later on, the pregnant females were randomly divided into two sets; one of them was injected intraperitoneally (i.p.) with a single dose of 0.9% saline and was considered as a control group, and the other animals were injected i.p. with sodium valproate (600 mg/kg, single dose) [30]. These injections were performed on the GD 12.5, after which the injected pregnant females were housed individually and left undisturbed till giving birth.

**Figure 1:**
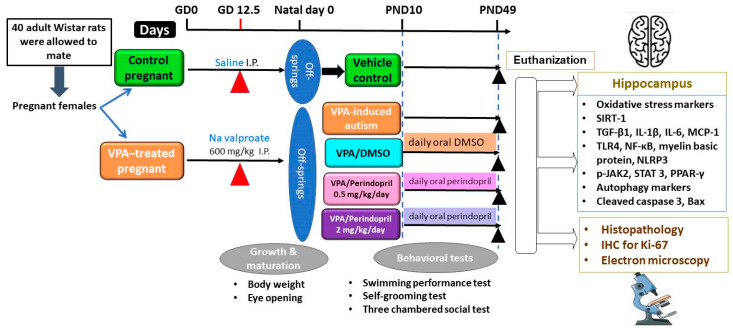
A timeline schematic diagram for the experimental design.

Weaning of the off-springs was carried out on postnatal day (PND) 21 and the male off-springs only were subjected to further experiments. Ten male off-springs born from the control pregnant females were assigned as the control group and received no treatment. Ten male off-springs born from valproate-treated mothers were considered as the VPA-induced autism group. Another ten male off-springs born from valproate-treated mothers were administered 0.25 mL of DMSO daily. Additionally, ten male pups from valproate-treated mothers were treated orally with a daily dose of perindopril (0.5 mg/kg) [31]. Ten male pups from valproate-treated mothers were treated orally with a daily dose of perindopril (2 mg/kg) [32]. Administration of DMSO and perindopril was carried out by oral gavage from the 10th PND to the 49th PND (Figure 1).

### 2.4. Determination of the Effect of Different Treatments on the Animals’ Postnatal Growth and Maturation

The weight of the animals was recorded at PND 7, 14, 21, 28, 35, 42, and 49. The animal’s maturation by daily observation of eye opening from the 12th PND to the 17th PND and scoring was performed as follows: 0 = both eyes closed, 1 = one eye opened, and 2 = both eyes opened [33].

### 2.5. Assessment of the Behavioral Changes in the Studied Groups

#### 2.5.1. Swimming Performance Test

This test was carried out to assess the motor development and the integration of reflex responses of rats exposed to the different treatments. It was performed on the 8th, 10th, 12th, 14th, and 16th PNDs where rats were allowed to swim at a specially prepared aquarium, swimming was observed for 10 s, and the performance of swimming was recorded and scored according to Zhou et al. [34].

#### 2.5.2. Self-Grooming Test

This test was performed to determine the repetitive behavior at the 49th PND. A standard cage of 25 cm width, 45 cm length, and 20 cm height was utilized in this test. Rats were kept in this cage for five minutes for habituation after which the cumulative grooming times spent for all body parts of each animal were measured using a stopwatch for five minutes and recorded [35].

#### 2.5.3. Three-Chambered Social Test

This test was carried out at the 49th PND to detect the changes in the social behavior of the tested animals. In this test, a box divided into three identical chambers with both side compartments containing an empty perforated cup was utilized. First, the entire box was thoroughly cleaned with 70% ethyl alcohol at the beginning of each trial. Then, habituation started by allowing the tested animal to explore the whole box freely, with all doors open for ten minutes. After that, the animal was kept in the central chamber, where a stranger rat 1 (an unfamiliar animal of the same gender) was put under one of the cups. Then, the tested animal was allowed to explore the whole box for another ten minutes after which it was kept again in the central chamber. Then, another stranger rat 2 (an unfamiliar rat of the same gender) was put under the other cup. The tested animal is freed again to explore the whole box for ten minutes. The time spent by the tested rat in each chamber was manually recorded and analyzed according to Rein et al. [36].

### 2.6. Determination of the Effect of Different Treatments on the Biochemical Parameters

#### 2.6.1. Tissue Collection and Processing

At the 50th PND, rats were anaesthetized with isoflurane and killed by cervical dislocation. The hippocampus was immediately extracted and washed with ice-cold saline. Parts of the hippocampus were homogenized using Teflon homogenizer and the crude homogenate was centrifuged at 3000× *g* r.p.m. for 15 min. The resulting supernatant was separated in Eppendorfs and further processed for quantification of the biochemical parameters. Other parts of the hippocampus were prepared for examination of the histopathological and electron microscopic changes.

#### 2.6.2. Assessment of the Redox Status and Sirtuin-1 (SIRT1) Levels in the Hippocampal Tissues

Kits purchased from Biohippo Inc., Gaithersburg, MD, USA (Catalog No. BHE13700861) were used for the assessments of MDA levels in the hippocampal tissues. Catalase (CAT), glutathione S-transferase (GST), and glutathione reductase (GR) levels were quantified in the hippocampal tissues using ELISA kits supplied by LSBio, Seattle, WA, USA (Catalog No. LS-F6440-1, LS-F40536-1, and LS-F6288-1, respectively). SIRT1 levels in the hippocampal tissues were determined by utilizing kits purchased from Biorbyt Ltd., Cambridge, CB4 0WY, UK (Catalog No. orb1196713).

#### 2.6.3. Quantification of the Hippocampal Tissue Content of Transforming Growth Factor Beta 1 (TGF-β1), Interleukin 1 Beta (IL-1β), IL-6, and Monocyte Chemoattractant Protein 1 (MCP-1)

Wuhan Fine Biotech Co., Wuhan, Hubei, China provided us with ELISA kits that were used for quantification of TGF-β1 and IL-1β levels (Catalog No. ER1378 and ER1094 respectively). IL-6 and MCP-1 were assessed in the hippocampal tissues using ELISA kits purchased from Boster Biological Technology, Pleasanton, CA, USA (Catalog No. EK0412 and EK0902, respectively).

#### 2.6.4. Measurement of the Hippocampal Tissue Content of Toll-like Receptor 4 (TLR4), Nuclear Factor Kappa B (NF-κB), Myelin Basic Protein, and Nod-like Receptor Protein 3 (NLRP3) Inflammasome

Biorbyt Ltd., Cambridge, UK was the provider of ELISA kits that were used for measurement of the hippocampal tissue content of TLR4 and myelin basic protein (Catalog No. orb410671 and orb1211781, respectively). NF-κB levels were quantified in the hippocampal tissues using kits supplied by ZellBio GmbH, Lonsee, Germany (Catalog No. RK03838). ABclonal, Woburn, MA, USA was the source of ELISA kits (Catalog No. RK04262) that were used for quantitative assessment of NLRP3 inflammasome levels in the hippocampal tissues.

#### 2.6.5. Determination of Phosphorylated Janus Kinase 2 (p-JAK2), Signal Transducer and Activator of Transcription-3 (STAT 3), and PPAR Gamma Levels in the Hippocampal Tissues

The hippocampal tissue levels of p-JAK2 and STAT3 were measured using kits purchased from MyBioSource, Inc., San Diego, CA, USA (Catalog No. MBS7269637 and MBS2515874, respectively). PPAR gamma levels in the hippocampal tissues were determined using kits supplied by CalibreScientific Global Distribution, Inc., Holland, OH, USA (Catalog No. RDB-RDR-PPARg-Ra-96T).

#### 2.6.6. Assessment of Phosphotylinosital-3-Kinase (PI3K)/Akt/Glycogen Synthase Kinase-3 (GSK-3β) Signaling Pathway in the Hippocampal Tissues

PI3K levels were determined in the hippocampal tissues using kits purchased from Biorbyt Ltd., Cambridge, UK (Catalog No. orb782226). The ratio of the levels of the phosphorylated form of Akt (pS473) to the total Akt levels in the hippocampal tissues was assessed using kits supplied by Thermo Fisher Scientific Inc., Branchburg, NJ, USA (Catalog No. 85-86046-11). Additionally, RayBiotech Life, Inc., Peachtree Corners, GA, USA provided the kits required for the determination of the ratio between the phosphorylated and the total forms of GSK-3β in the hippocampal tissues (Catalog No. PEL-GSK3b-S9-T-1).

#### 2.6.7. Quantification of the Hippocampal Tissue Content of Autophagy Markers

The hippocampal tissue levels of LC3-II levels were quantified using ELISA kits obtained from MyBioSource, Inc., San Diego, CA, USA (Catalog No. MBS1600540). Additionally, RayBiotech Life, Inc., Peachtree Corners, GA, USA provided the kits required for assessment of the ratio between the phosphorylated and the total forms of beclin-1 levels in the hippocampal tissues (Catalog No. PEL-BECLIN1-S234-T-1).

#### 2.6.8. Assessment of the Apoptotic Changes in the Hippocampal Tissues

Cleaved caspase 3 levels were measured in the hippocampal tissues using kits obtained from Sunlong Biotech, Hangzhou, Zhejiang, China (Catalog No. SL1366Ra). BioVision, Inc., Waltham, MA, USA supplied ELISA kits that were used for quantification of Bax levels in the hippocampal tissues (Catalog No. E4513).

### 2.7. Evaluation of the Histopathological Changes Induced by Different Treatments in the Hippocampal Tissues

Parts of the right hippocampal hemisphere were immediately immersed in 10% formaldehyde solution for 24 h, after which they were prepared to form paraffin blocks. These blocks were cut with a microtome (Leica Biosystems, Deer Park, IL, USA) at 5 microns thickness, then left to dry overnight on glass slides in the oven at 37 °C. Then, these slides were immersed in xylene for 15 min after which they were put in four changes of absolute alcohol then immersed in descending grades of alcohol. After that, staining with hematoxylin and eosin (H&E) took place for further examination under a light microscope (Olympus, Tokyo, Japan). Counting of the numbers of Purkinje cells in the hippocampal hemisphere was performed using ImageJ software (Version 1.52f), National Institutes of Health at 20× magnification.

### 2.8. Detection of the Immunoexpression of Ki-67 in the Hippocampal Tissues

Sections from the hippocampal tissues were stained for immunohistochemical examination using the three steps indirect streptavidin method that utilizes Ki-67 monoclonal antibody (SolA15) (Thermo Fisher Scientific, USA, Catalog No. 11-5698-82). The primary antibody for Ki-67 was omitted under identical test conditions to obtain the technical negative controls. Ki-67 positive staining was identified in the hippocampal tissues as brown nuclear staining with a diffuse pattern. The expression of Ki-67 was assessed semi-quantitatively and scored by counting at least 1000 cells in ten high-power fields. The percentage of the positively-stained cells was regarded as the Ki-67 labeling index [37].

### 2.9. Assessment of the Electron Microscopic Changes in the Hippocampal Tissues

Specimens from the hippocampus were immersed in a mixture of 2% paraformaldehyde and 2.5% glutaraldehyde in a 0.1 M cacodylate buffer (pH 7.4). After that, these specimens were fixed in an ice-cold fixative solution for 20 h and put in a mixture of 1% OsO4 and 0.8% K_4_[Fe(CN)_6_]. Then, these specimens were dehydrated in a series of different concentrations of ethanol which later on were embedded in epoxy resin. The ultramicrotome was used to obtain ultra-thin sections of the hippocampal tissues of 60 nm thickness which were then examined by transmission electron microscope (JEM-1200EX, Jeol, Tokyo, Japan). Electronograms under a magnification of 50,000× were obtained for assessment of the number of the synaptic vesicles in the nerve endings.

### 2.10. Statistical Evaluation of the Obtained Data

Using GraphPad Prism software, version 9 (La Jolla, CA, USA), the data obtained from the current study were statistically analyzed and displayed as mean ± standard deviation (SD). Specifically, a one way-analysis of variance (ANOVA) test followed by a post-hoc Tukey test were used to detect the differences between the different groups. Data regarding the behavioral tests and eye-opening were statistically analyzed using non-parametric tests including the Kruskal–Wallis and Mann–Whitney test. The degree of statistical significance was acceptable when *p*-values were less than 0.05.

## 3. Results

### 3.1. Perindopril Restored the Growth and Maturation Rates of Rats Exposed Prenatally to VPA

**Figure 2:**
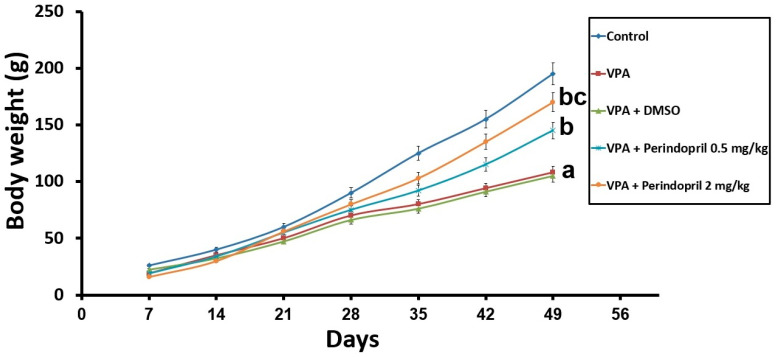
Effect of the different treatments on the body weight gain (^a^ significant VS the control group; ^b^ significant VS sodium valproate group; ^c^ significant VS sodium valproate group treated with 0.5 mg/kg/day perindopril).

**Figure 3:**
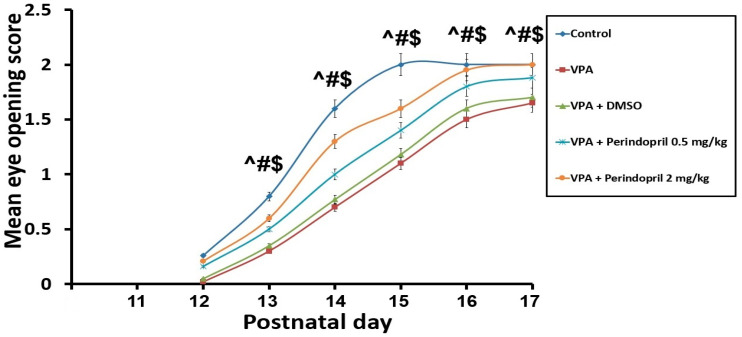
Effect of the different treatments on the mean eye-opening score (^ sodium valproate group VS the control group; # Perindopril groups VS sodium valproate group; $ The group treated with 2 mg/kg/day perindopril VS the group treated with 0.5 mg/kg/day perindopril).

Animals subjected to VPA prenatally exhibited retarded maturation, evidenced by a significant reduction in the body weight gain (*p* = 0.003) and delayed eye-opening (*p* < 0.001) relative to animals of the control group. Administration of DMSO to the animals prenatally subjected to VPA did not significantly affect their growth and maturation rates relative to the untreated rats exposed prenatally to VPA (*p* = 0.09). However, administration of perindopril dose-dependently induced restoration of the body weight gain (*p* < 0.001) with a significant increase in the eye-opening scores (*p* = 0.002) relative to rats exposed to VPA alone in the prenatal period (Figure 2 and Figure 3).

### 3.2. Perindopril Restored the Outcomes of the Behavioral Tests to the Reference Values in Rats Exposed Prenatally to VPA

**Figure 4:**
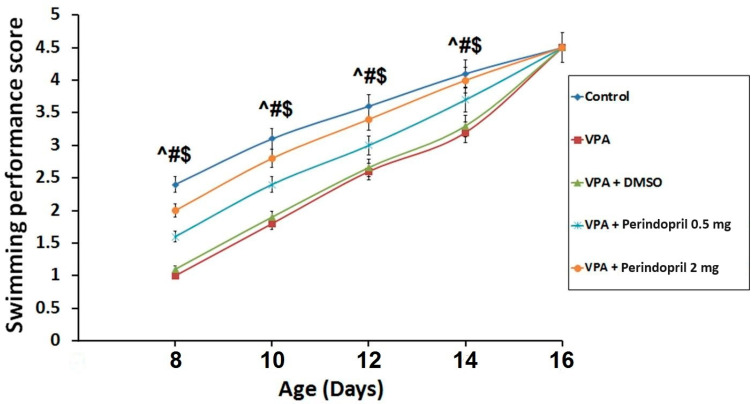
Swimming performance score in the studied groups (^ sodium valproate group VS the control group; # Perindopril groups VS sodium valproate group; $ The group treated with 2 mg/kg/day perindopril VS the group treated with 0.5 mg/kg/day perindopril).

In the untreated rats exposed prenatally to VPA, there was significant delay in the swimming behavior relative to animals of the control group (*p* < 0.001). Administration of DMSO did not significantly affect the swimming behavior relative to the untreated rats exposed prenatally to VPA (*p* > 0.05). Rats treated with perindopril exhibited dose-dependent improvement of the swimming performance relative to the untreated rats exposed prenatally to VPA (*p* < 0.001) (Figure 4).

**Figure 5:**
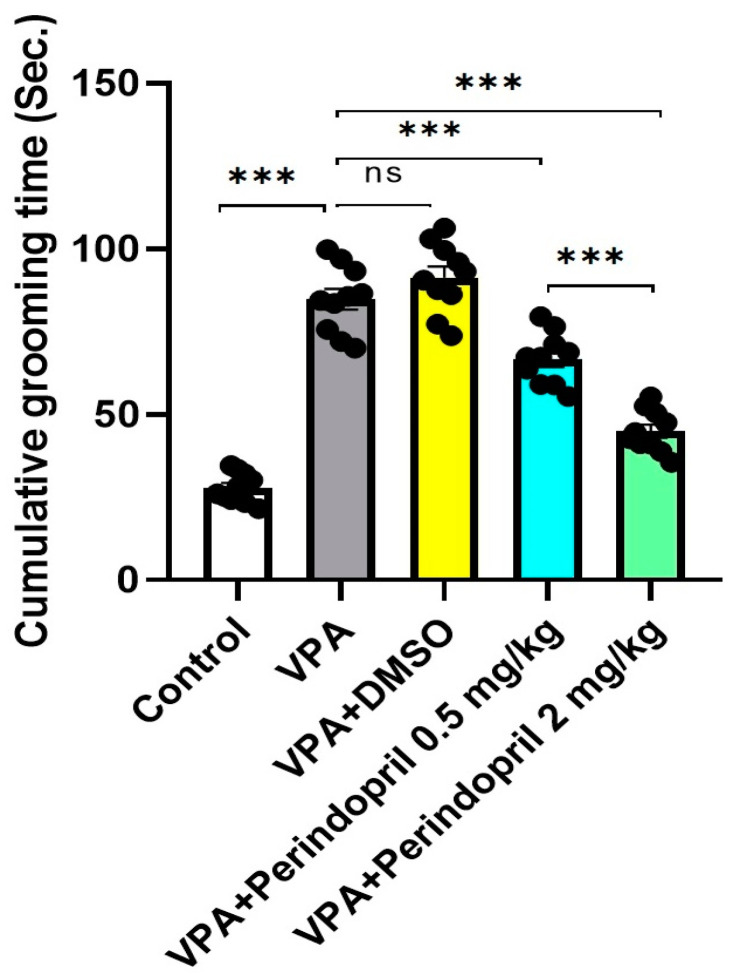
Self-grooming test in the studied groups (ns = non-significant; *** = *p* < 0.001).

When compared versus the control group, the untreated rats exposed prenatally to VPA exhibited significant prolongation of the cumulative self-grooming time (*p* < 0.001). This prolongation was not significantly affected by administration of DMSO to rats exposed prenatally to VPA (*p* > 0.05). Interestingly, this prolongation was dose-dependently shortened with administration of perindopril (*p* < 0.001), thereby indicating its role in normalization of the repetitive behavior (Figure 5).

**Figure 6:**
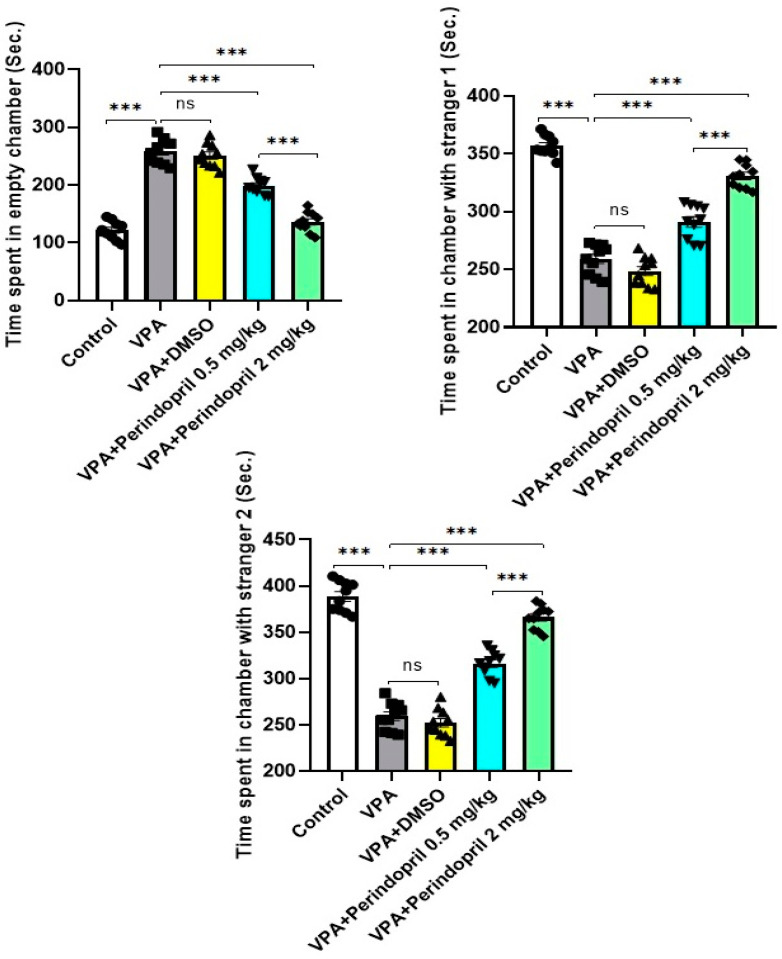
Three-chambered social test in the studied groups (ns = non-significant; *** = *p* < 0.001).

As shown in Figure 6, the untreated rats exposed prenatally to VPA exhibited significantly disturbed social behavior manifested by the presence of non-significant differences between the time spent by the tested animals in the empty chamber relative to that spent in the chamber containing stranger 1 (*p* = 0.11). Dose-dependent prolongation of the time spent by the tested animals in the chamber containing stranger 1 relative to the time spent in the empty chamber was observed in the groups treated with perindopril and indicated significant improvement of sociability.

Significant diminution in the preference for social novelty and significant affection of the social interaction behaviors were demonstrated in Figure 6 where the untreated rats exposed prenatally to VPA did not show significant preference for the chamber containing the newly introduced stranger 2 relative to the chamber containing the familiar stranger 1 (*p* = 0.09). Animals treated with perindopril exhibited dose-dependent significant prolongation of the time spent by the tested animals in the chamber containing the newly introduced stranger 2 relative to that spent in the chamber containing the familiar stranger 1. Administration of DMSO did not significantly affect any of the social behaviors relative to the untreated rats exposed prenatally to VPA (*p* > 0.05).

### 3.3. Perindopril Restored the Normal Values of SIRT1 and the Redox Status in the Hippocampal Tissues of Rats Prenatally Exposed to VPA

**Figure 7:**
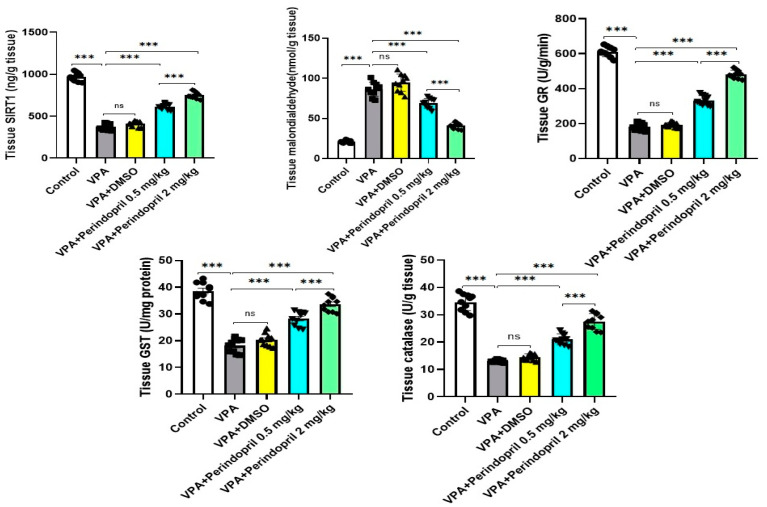
Effect of the different doses of perindopril on SIRT1 levels and the redox status of the hippocampal tissues of rats exposed prenatally to sodium valproate (*** = *p* < 0.001, ns = non-significant).

As depicted in Figure 7, prenatal exposure to VPA augmented oxidative stress in the hippocampal tissues, as evidenced by significant elevation in tissue MDA levels (*p* < 0.001) and significant decrement in the tissue levels of SIRT1 (*p* < 0.001) and antioxidant enzymes (*p* < 0.001) when compared to the respective values detected in animals born to the control mothers. Administration of DMSO to the animals prenatally subjected to VPA did not significantly affect the tissue redox status (*p* > 0.05) and SIRT1 levels (*p* = 0.18) relative to the untreated rats exposed prenatally to VPA. Interestingly, perindopril dose-dependently had the ability to reverse these changes with a significant decrease in MDA levels (*p* < 0.001) and significant elevation in SIRT1 (*p* < 0.001) and antioxidant enzymes levels (*p* < 0.001) in the hippocampal tissues when compared versus rats prenatally exposed to VPA alone.

### 3.4. Perindopril Abrogated the Effect of VPA Prenatally on the Hippocampal Tissue Content of TGF-β1, IL-1β, IL-6, and MCP-1

**Figure 8:**
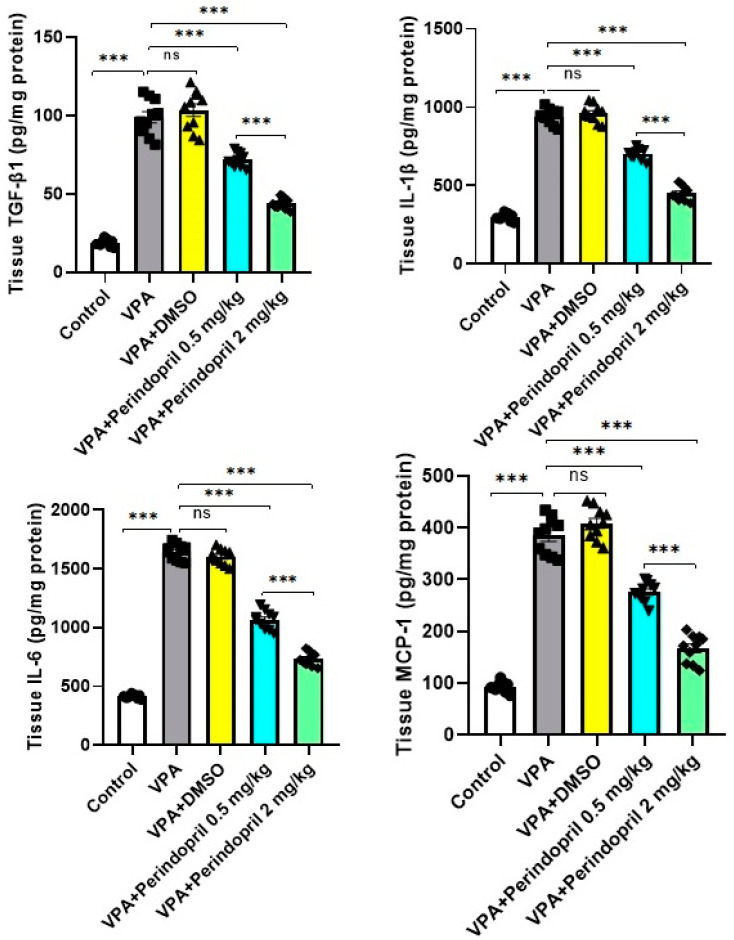
Effect of the different doses of perindopril on TGF-β1, IL-1β, IL-6, and MCP-1 levels in the hippocampal tissues of rats exposed prenatally to sodium valproate (*** = *p* < 0.001, ns = non-significant).

As demonstrated in Figure 8, significant elevation in the hippocampal tissue levels of TGF-β1 (*p* < 0.001), IL-1β (*p* < 0.001), IL-6 (*p* < 0.001), and MCP-1 (*p* < 0.001) was detected in rats exposed prenatally to VPA relative to the control group. No significant changes in the aforementioned biochemical measurements were detectable with the administration of DMSO to rats exposed prenatally to VPA (*p* > 0.05). Meanwhile, perindopril expressed a dose-dependent ability to ameliorate the changes in the afore-mentioned parameters induced by prenatal exposure to VPA (*p* < 0.001).

### 3.5. Perindopril Mitigated the Effects of VPA Prenatally on the Hippocampal Tissue Content of TLR4, NF-κB, Myelin Basic Protein, and NLRP3 Inflammasome

**Figure 9:**
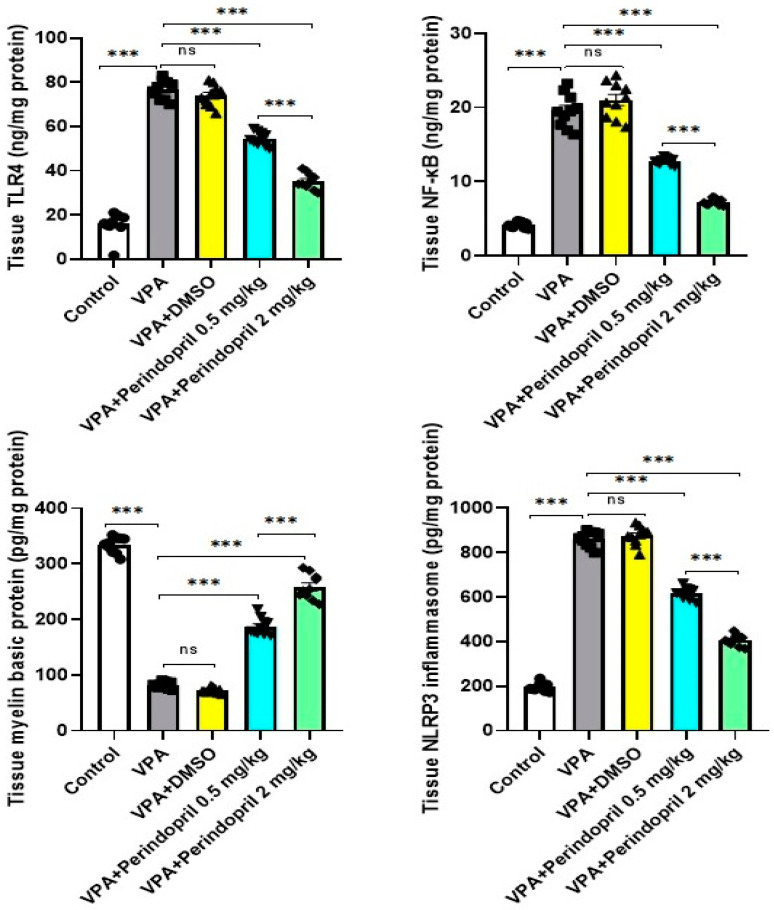
Effect of the different doses of perindopril on TLR4, NF-κB, myelin basic protein, and NLRP3 inflammasome levels in the hippocampal tissues of rats exposed prenatally to sodium valproate (*** = *p* < 0.001, ns = non-significant).

Significant elevation of the hippocampal tissue levels of TLR4 (*p* < 0.001), NF-κB (*p* < 0.001), and NLRP3 inflammasome (*p* < 0.001) associated with a significant decline in the hippocampal tissue levels of myelin basic proteins (*p* < 0.001) were obviously observed in animals prenatally exposed to VPA when compared versus the control group. Administration of DMSO did not restore the levels of these parameters to approximate the normal levels (*p* > 0.05). Nevertheless, administration of perindopril dose-dependently reversed the aforementioned changes relative to those detected in the untreated rats exposed prenatally to VPA (*p* < 0.001) (Figure 9).

### 3.6. Perindopril Impeded the Changes Induced by VPA Prenatally in p-JAK2/STAT3/PPAR Gamma Signaling in the Hippocampal Tissues

**Figure 10:**
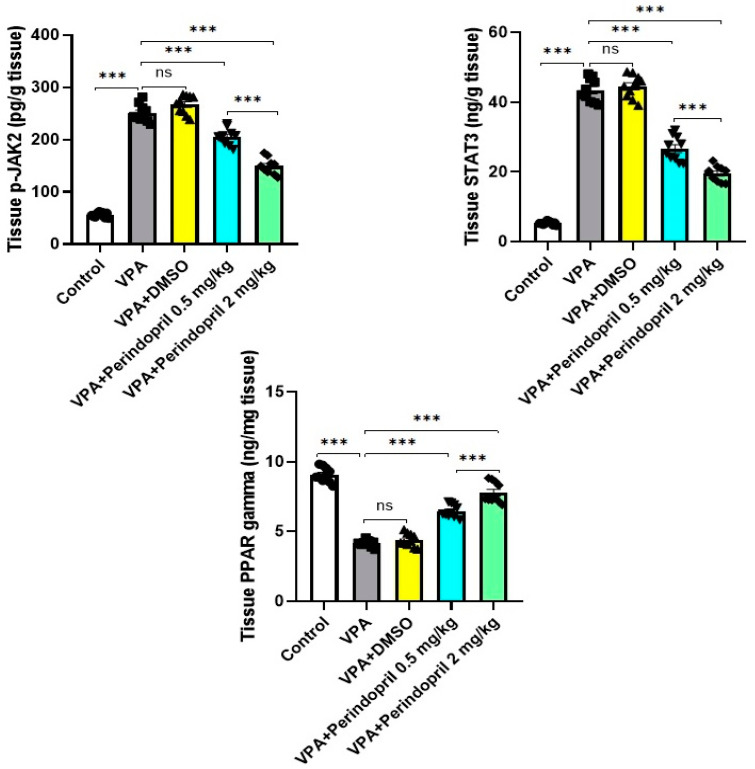
Effect of the different treatments on p-JAK2, STAT3, and PPAR gamma levels in the hippocampal tissues (*** = *p* < 0.001, ns = non-significant).

The results obtained from the present study revealed that VPA prenatally elicited significant enhancement of *p*-JAK2/STAT3 signaling (*p* < 0.001) associated with a significant decrease in PPAR gamma signaling (*p* < 0.001) in the hippocampal tissues relative to the control rats. Although treatment of VPA-exposed animals with DMSO did not significantly counteract the effects of VPA prenatally on the aforementioned parameters (*p* > 0.05), perindopril exhibited dose-dependent ameliorative effects on the *p*-JAK2/STAT3 pathway (*p* < 0.001) together with augmentation of PPAR gamma signaling in the hippocampal tissues (*p* < 0.001) when compared versus the untreated VPA-exposed animals (Figure 10).

### 3.7. Perindopril Curtailed the Changes in PI3K/Akt/GSK-3β Pathway in the Hippocampal Tissues Elicited by Prenatal Exposure to VPA

**Figure 11:**
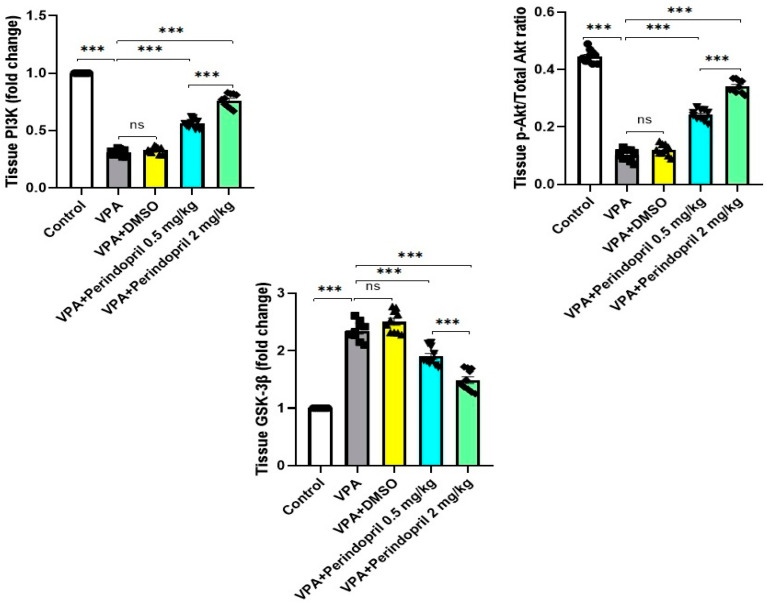
Effect of the different treatments on the PI3K/Akt/GSK-3β axis in the hippocampal tissues (*** = *p* < 0.001, ns = non-significant).

As depicted in Figure 11, prenatal exposure to VPA significantly affected PI3K/Akt/GSK-3β signaling in the form of significant decline in PI3K (*p* < 0.001) and *p*-Akt/total Akt levels (*p* < 0.001) with significant elevation in GSK-3β expression in the hippocampal tissues (*p* < 0.001) relative to the control group. These changes were reversed with the administration of perindopril, in a dose-dependent manner, when compared to rats prenatally exposed to VPA (*p* < 0.001). Treatment of VPA-exposed rats with DMSO did not significantly affect PI3K/Akt/GSK-3β signaling when compared to the untreated rats exposed prenatally to VPA (*p* > 0.05).

### 3.8. Perindopril Curbed the Effects of VPA Prenatally on the Autophagy Markers in the Hippocampal Tissues

**Figure 12:**
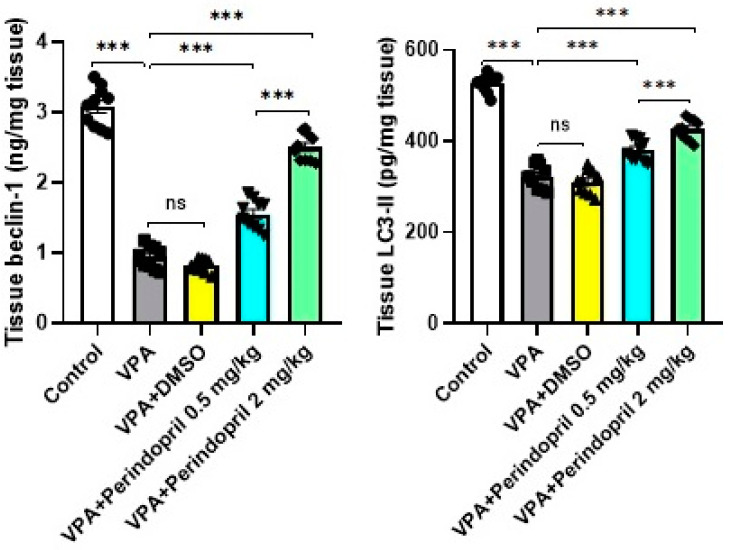
Effect of the different treatments on beclin-1 and LC3-II levels in the hippocampal tissues (*** = *p* < 0.001, ns = non-significant).

Figure 12 demonstrated the effect of different treatments on the autophagy markers. The significant decrease in the hippocampal tissue content of beclin-1 (*p* = 0.002) and LC3-II levels (*p* < 0.001) induced by VPA prenatally relative to the control group was dose-dependently mitigated with the administration of perindopril (*p* < 0.001). However, treatment of rats prenatally exposed to VPA with DMSO did not exhibit significant changes in the hippocampal tissue levels of beclin-1 (*p* = 0.16) and LC3-II (*p* = 0.21) relative to the untreated VPA-exposed group.

### 3.9. Perindopril Attenuated the Pro-Apoptotic Changes in the Hippocampal Tissues Created by Prenatal Exposure to VPA

**Figure 13:**
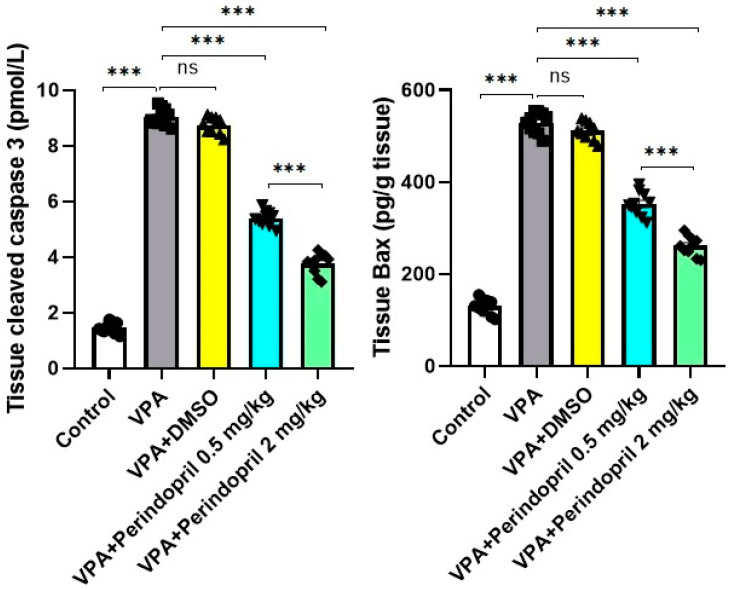
Effect of the different treatments on cleaved caspase 3 and Bax levels in the hippocampal tissues (*** = *p* < 0.001, ns = non-significant).

As shown in Figure 13, prenatal exposure to VPA elicited significant elevation of cleaved caspase 3 (*p* < 0.001) and Bax levels (*p* = 0.004) in the hippocampal tissues relative to the control group. Although the treatment of VPA-exposed rats with DMSO did not affect these apoptotic markers (*p* > 0.05), perindopril dose-dependently ameliorated these apoptotic changes with significant decline in tissue cleaved caspase 3 (*p* < 0.001) and Bax levels (*p* < 0.001) when compared versus the untreated VPA-exposed group.

### 3.10. Perindopril Counteracted the Effects of VPA Prenatally on the Histopathological Picture of the Hippocampal Tissues

**Figure 14:**
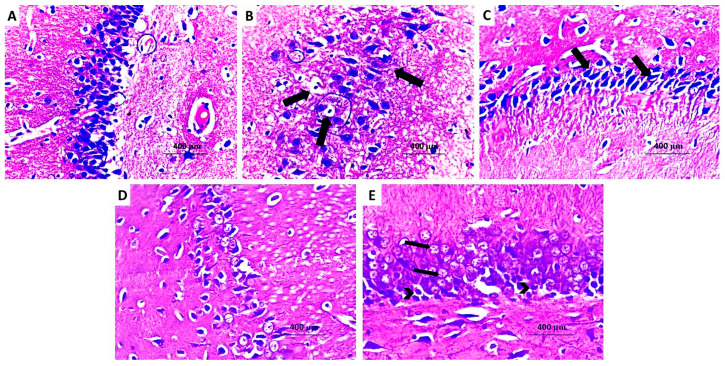
A photomicrograph of H&E stained sections from the hippocampus of (**A**) the control group with regular adherent arrangement of the pyramidal cells with normal distribution of the polygonal cell bodies, vesicular nuclei, and prominent nucleoli (H&E ×400). (**B**) Sodium valproate group with disrupted arrangement of the pyramidal cells, evident decrease in the pyramidal cell mass, dystrophic neurons, shrunken hyperchromatic pyknotic nuclei, and condensed chromatin (H&E ×400). (**C**) Sodium valproate group treated with dimethyl sulfoxide showing dystrophic apoptotic neurons with shrunken hyperchromatic pyknotic nuclei and condensed chromatin (H&E ×400). (**D**) Sodium valproate group treated with 0.5 mg/kg/day perindopril showing moderate decrease in the number of the apoptotic neurocytic nuclei with evident increase in the number of the normal nuclei (H&E ×400). (**E**) Sodium valproate group treated with 2 mg/kg/day perindopril showing increased number of the normal neurons (Arrows) and marked decrease in the number of the apoptotic neurons (Arrow heads) (H&E ×400).

The hippocampal tissues of the control group showed compact layers of small and large pyramidal cells with polygonal cell bodies, vesicular nuclei, and prominent nucleoli (Figure 14A). Both sodium valproate and sodium valproate + DMSO groups showed decreased thickness of the pyramidal cell layer, an increased number of the dystrophic apoptotic neurons, shrunken hyperchromatic pyknotic nuclei, and condensed chromatin (Figure 14B,C, respectively). Treatment with perindopril revealed a dose-dependent decrease in the number of the apoptotic neurocytic nuclei associated with a noticeable increase in the number of the normal nuclei (Figure 14D,E).

### 3.11. Perindopril Induced Significant Detrimental Effects on Ki-67 Immunoexpression in the Hippocampal Tissues of Rats Prenatally Exposure to VPA

**Figure 15:**
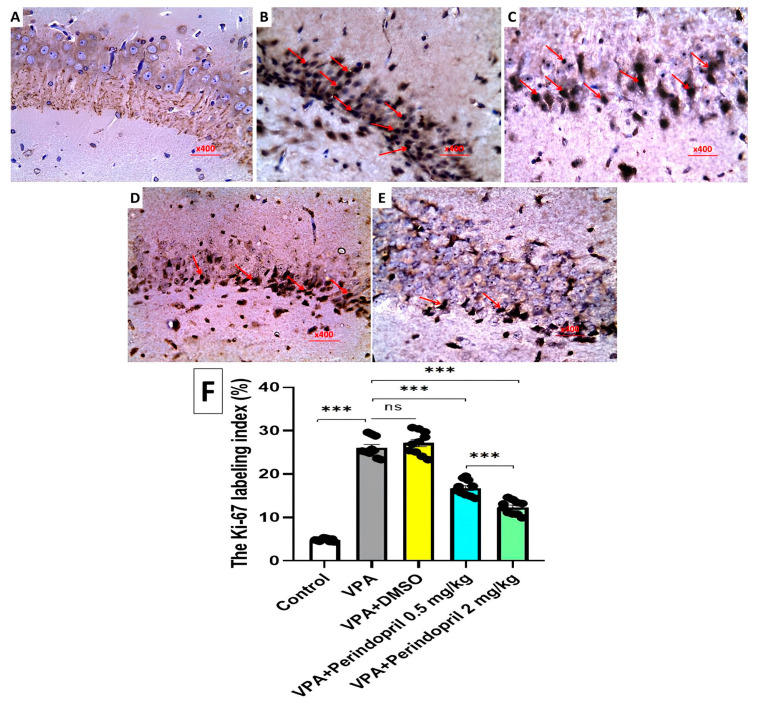
A photomicrograph of Ki-67 stained sections (Streptavidin biotin × 400) of the hippocampus of (**A**) the control rats showing negative immune reactivity of the pyramidal cells to Ki-67. (**B**,**C**) Sodium valproate and sodium valproate + DMSO-treated groups, respectively, showing strongly positive immunostained apoptotic nuclei (Arrows). (**D**) Sodium valproate group treated with 0.5 mg/kg/day perindopril showing decreased immune reactivity of the neurocytic nuclei to Ki-67 (moderate expression) (Arrows) with a noticeable increase in number of the negatively stained normal nuclei. (**E**) Sodium valproate group treated with 2 mg/kg/day perindopril showing marked decrease in immune reactivity (weak expression) of the neurocytic nuclei to Ki-67 (Arrows). (**F**) The Ki-67 labeling index (*** = *p* < 0.001, ns = non-significant).

In rats prenatally exposed to VPA, the hippocampal tissues exhibited strong positive Ki-67 immunostaining relative to the control group (Figure 15A,B,F). Treatment with DMSO did not significantly modulate the extent of Ki-67 immunostaining relative to the untreated VPA-exposed group (Figure 15C,F). VPA-exposed animals treated with perindopril exhibited a dose-dependent decline in tissue Ki-67 immunostaining relative to the untreated VPA-exposed animals (Figure 15D–F).

### 3.12. Perindopril Significantly Mitigated the Electron Microscopic Changes in the Hippocampal Tissues Elicited by Prenatal Exposure to VPA

**Figure 16:**
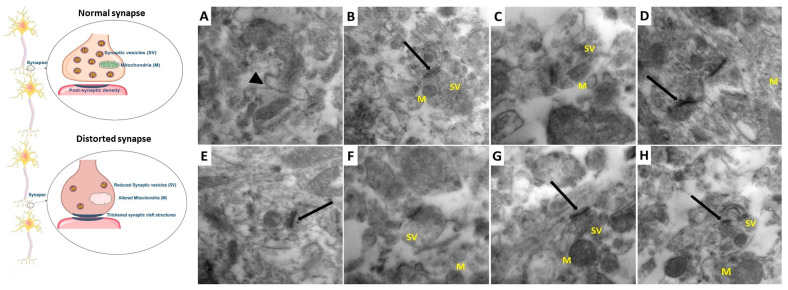
Electron micrographs of ultrathin sections in the hippocampus from rats of (**A**,**B**) the control group showing normal appearance of the synaptic cleft (Arrow head), well-defined structure of the synapses with accurate postsynaptic density (Long arrow), normal distribution of the synaptic vesicles (SVs), and normally-shaped mitochondria with normal cristae pattern (M) appearing within their axoplasm. (**C**,**D**) Sodium valproate group showing reduced packing density of the synaptic vesicles (SVs) in the presynaptic area with swelling of the nerve endings, blurred and thickened structure of the synaptic cleft without clear marked pre- and post-synaptic membranes (Long arrow), and ultra-structurally changed mitochondria with a blurred cristae structure (M). (**E**,**F**) Sodium valproate group treated with DMSO showing scanty distribution of the synaptic vesicles (SVs) in the presynaptic area, marked increase in the post-synaptic density of the synaptic cleft with blurred pre- and post-synaptic membranes (Long arrow), and dilated mitochondria with disrupted cristae (M). (**G**) Sodium valproate group treated with 0.5 mg/kg/day perindopril showing moderate distribution of the synaptic vesicles (SVs) in the presynaptic area, improvement of the post-synaptic density of the synaptic cleft with clearly marked pre- and post-synaptic membranes (Long arrow), and dilated mitochondria with preserved cristae (M). (**H**) Sodium valproate group treated with 2 mg/kg/day perindopril showing normal distribution of the synaptic vesicles (SVs) in the presynaptic area, well-defined structure of the synapses with accurate postsynaptic density (Long arrow), and normally distributed mitochondria with normal cristae pattern (M). The illustrations were constructed using SMART SERVIER MEDICAL ART at https://smart.servier.com/, the accessed date was 18 May 2023.

As depicted in Figure 16C,D, rats treated with sodium valproate alone showed a significant reduction in the packing density of the synaptic vesicles in the presynaptic area with swelling of the nerve endings, blurred and thickened structure of the synaptic cleft without clear marked pre- and post-synaptic membranes, and ultra-structurally changed mitochondria with a blurred cristae structure (M). Administration of DMSO did not significantly influence the electron microscopic changes induced by sodium valproate administration (Figure 16E,F). Administration of perindopril dose-dependently combatted the electron microscopic changes induced by sodium valproate administration, manifested by restoration of the normal distribution of the synaptic vesicles in the presynaptic area, improvement of the post-synaptic density of the synaptic cleft with clearly marked pre- and post-synaptic membranes, and restoration of the normal architecture and distribution of the mitochondria (Figure 16G,H).

## 4. Discussion

Autism spectrum disorder includes a wide range of neurodevelopmental disabilities characterized by marked affection of the social skills and abnormal repetitive behaviors, speech, and communication [38]. Many subtypes of autism spectrum disorder are recognized which are determined by a combination of genetic and environmental elements [39]. The exact etiology of this disorder is not yet fully explored, although affection of the redox status and the inflammatory cascade in the hippocampal tissues together with disruption of the balance between the autophagic and the proapoptotic signals are recognized by recent research as strong contributing factors [40]. This was in agreement with the findings encountered in the present study where rats prenatally exposed to VPA exhibited significant affection of the growth, maturation, motor development, and repetitive and social behaviors relative to the control group. VPA was used in the present study to induce a model of autism based on previous reports that indicated that the autism spectrum disorder induced by VPA has a close similarity to the human data concerning autism [41]. Qi et al. [42] attributed the delayed maturation and motor development together with the incoordination of reflexes induced by prenatal exposure to VPA to its ability to affect the synaptic activity with modulation of the neuronal excitability. The social deficits and the repetitive behaviors elicited by prenatal exposure to VPA were thought to be due to interference with GABAergic signaling with subsequent disruption of the balance between the excitatory and the inhibitory neurotransmitters in the brain leading to delayed neural circuits with the net result of impairment of the social interactions and communication with increased repetitive behaviors [43].

In the present study, the hippocampus was selected for molecular, histopathological, and electron microscopic analysis due to its critical role in the pathogenesis of autism phenotype [44]. Autism is characterized by impaired social communication and repetitive behavior in addition to difficulties involving cognition, memory, learning, language ability, emotions, and creation of the cognitive map [45]. The hippocampus was proven to play a critical role in learning, memory, spatial reasoning, perception of emotions to others, and elaboration of reciprocal social interactions which are impaired in patients with autism [46]. Moreover, the ongoing research showed structural and functional changes in the hippocampus more than other regions of the brain in children with autism [47]. These changes may be related to altered blood flow, metabolism, morphology, and neuroimmunity and impairment of the connectivity to other brain areas [48]. Furthermore, there is a strong evidence that social and behavioral deterioration occurs between the age of one-and-a-half to two years which is the same period related to the major hippocampal developmental milestones and responsible for connections between the hippocampus and the other important areas in the brain [49]. So, the study of the hippocampus is very important in autism research due to the strong evidence of its relation to the pathogenesis of autism. SIRT1 protein is nicotinamide adenine dinucleotide dependent histone deacetylase that functions as a transcription factor for a wide range of physiological processes in the different body systems [50]. Recent reports suggest that there is a close relationship between SIRT1 levels in the cerebral tissues and the pathogenic events that occur in autism [43]. Bu et al. [51] stated that SIRT1 combats oxidative stress in the neuronal tissues possibly via enhancing the expression of peroxisome proliferator-activated receptor-γ co-activator 1α (PGC-1α) with subsequent inhibition of ROS production. In addition, SIRT1 enhances the expression of the antioxidant enzymes via the FoxO pathway [52]. Moreover, SIRT1 was proven to inhibit the inflammatory responses in the neural tissues, an action that is mediated via modulation of NLRP3 inflammasome and NF-κB signaling [53]. The increased level of ROS was reported to suppress SIRT1 activity by oxidative modification of its cysteine residues [52]. This coincided with the findings of the present study where rats prenatally exposed to VPA exhibited a significant decrease in SIRT1 levels which was associated with significant deterioration of the antioxidant defense mechanisms and significant enhancement of the inflammatory cascade relative to the control group. These changes were reversed with the administration of perindopril which was in line with Zhu et al. [54] who reported that perindopril increases the level of SIRT1 with subsequent enhancement of the antioxidant and the anti-inflammatory pathways.

Accumulating data had proven that the crosstalk between TLR4, NF-κB, and NLRP3 inflammasome is the keystone of the pathogenic events that occur in autism [55,56]. Malaguarnera et al. [57] reported that the neuroinflammation induced by prenatal exposure to VPA in autism spectrum disorders increases the hippocampal tissue levels of TLR4 and NF-κB which enhance the progression of the pathogenic events of autism via increased expression of TGF-β1, MCP-1, and the proinflammatory cytokines. This inflammatory storm in the hippocampal tissues was proven to be associated with the activation of NLRP3 inflammasome with subsequent enhancement of caspase 1 expression with activation of the apoptotic pathways in the hippocampal tissues [58,59]. Agents that interfere with TLR4/NF-κB/NLRP3 inflammasome signaling in the hippocampal tissues were proven to significantly mitigate the neuroimmune dysregulation frequently encountered in autism [60]. These reports coincided with the findings of the present study where prenatal exposure to VPA elicited significant elevation of the hippocampal tissue levels of TLR4, NF-κB, NLRP3 inflammasome, Ki-67, TGF-β1, MCP-1, and the proinflammatory cytokines associated with significant apoptotic changes relative to the control group. Interestingly, these changes were dose-dependently ameliorated with the administration of perindopril. This was in line with the reports by El-Shoura et al. [61] who attributed the anti-neuroinflammatory effects of perindopril to inhibition of the TLR4/NF-κB axis. In addition, Zhang et al. [62] stated that perindopril interferes with the pathways of activation of NLRP3 inflammasome, minimizing the levels of the proinflammatory cytokines, and enhancing the antiapoptotic signaling pathways.

Myelin basic protein is one of the most prevalent proteins in the myelin sheath of the central nervous system [63]. It helps with adhesion of the cytosolic surfaces of multilayered compact myelin, thus playing a role in neurotransmission [64]. The current study demonstrated a significant decrease in myelin basic proteins levels in the hippocampal tissues of rats prenatally exposed to VPA. This coincided with Graciarena et al. [65] who stated that VPA administration elicited alterations in oligodendroglial-lineage cells and induced hypomyelination which may be considered as the neuropathological hallmarks of autism. Moreover, Gonzalez-Gronow et al. [66] explained the significant decrease in the tissue levels of myelin basic proteins in cases with autism by the development of catalytic autoantibodies to these proteins with the net result of hypomyelination and significant affection of neurotransmission. In agreement with our findings, Sayed et al. [28] reported that the anti-inflammatory effects of perindopril may be via targeting the extracellular signal-regulated kinase (ERK) and interference with the nuclear translocation of c-Fos, a subunit of the activator protein (AP)-1.

Accumulating evidence highlighted the role played by the JAK2/STAT3 axis and PPAR-gamma signaling in the pathogenesis of autism spectrum disorders [11]. Upon activation by the proinflammatory cytokines released in cases with autism, JAK2 receptors were proven to enhance STAT3 phosphorylation in the astrocytes and microglia [67,68]. Consequently, this phosphorylation initiated certain cellular events that lead to profound mitochondrial damage, activation of the apoptotic pathways, wide-spread neuroinflammation, and induction of reactive gliosis [11]. Interestingly, PPAR gamma was proven to effectively modulate the JAK2/STAT3 axis in the neurological tissues [69]. Agents that increase PPAR gamma levels in the brain were reported to combat the inflammatory cascade via inhibiting JAK 1, 2, STAT 1, and 3 phosphorylation [11]. Endogenous PPAR gamma ligands were proven to suppress chemokine protein production induced by interferon gamma and abrogate the JAK/STAT signaling pathway [70]. This was in line with the findings of the present study where prenatal exposure to VPA enhanced JAK2/STAT3 signaling and inhibited the expression of PPAR gamma in the hippocampal tissues relative to the control group. However, administration of perindopril in the current work dose-dependently increased the hippocampal tissue levels of PPAR gamma which was able to abrogate the phosphorylation and inhibit the activity of JAK2/STAT3 signaling pathway. These effects were supported by Şen and Hacıosmanoğlu [27] who demonstrated that perindopril exerted potent anti-inflammatory effects in the brain tissues via upregulation of PPAR gamma which have potent inhibitory effects on the different levels of the inflammatory cascade. Moreover, El-Shoura et al. [71] threw light on the JAK2/NF-κB p65 pathway as a potential target for the anti-inflammatory effect of perindopril.

Coinciding with the data obtained from the present study, the results of the recent research have shone a light on the crucial role played by PI3K/Akt/GSK-3β signaling in the pathogenesis of autism [13]. Prenatal exposure to VPA was reported to mitigate PI3K/Akt -mediated autophagic activity with the disappearance of the autophagosomes in the hippocampal tissues [72]. Subsequently, GSK-3β was freed from the inhibitory effect exerted by PI3K/Akt signaling resulting in the activation of the GSK-3β/β-catenin pathway with the net result of massive affection of the hippocampal neurons [14,73]. Amazingly, perindopril in the current work elicited a significant increase in PI3K and p-Akt/total Akt levels with significant decrement in GSK-3β expression in the hippocampal tissues when compared versus the VPA group. Similar data were demonstrated by Zakaria et al. [74] who reported that targeting the PI3K/Akt/GSK-3β axis by perindopril can efficiently ameliorate a wide range of inflammatory disorders, possibly through restoration of autophagy and mitigation of the inflammatory response.

In autism spectrum disorder, disturbed autophagy/apoptosis balance was regarded as the key event that is responsible for the main pathognomonic features of autism [75]. In agreement with the results of the current work, VPA was reported to initiate the apoptotic cascade in the hippocampal tissues by activation of caspase 1 via mechanisms related to NF-κB/NLRP3 inflammasome signaling [76]. At the same time, significant affection of PI3K/Akt/mTOR-mediated autophagy was proven to significantly affect the viability of the hippocampal neurons with subsequent deterioration of motor development and social behavior [72]. Interestingly, the results of the current work have confirmed the hypothesis of Zhu et al. [54] who stated that the beneficial effects of perindopril can be attributed, at least in part, to its ability to restore autophagy/apoptosis balance in the different body tissues.

## 5. Conclusions

The data herein presents perindopril as a promising agent for amelioration of the pathogenic changes of autism spectrum disorders. This may be attributed to its dose-dependent modulatory effects on sirtuin-1, oxidative stress, PI3K/Akt/GSK-3β pathway, JAK2/STAT3 axis, and PPAR-gamma signaling in addition to its potent anti-inflammatory and antiapoptotic effects. Further studies that shed light on the molecular mechanisms by which perindopril produce these effects and properly evaluate the clinical significance of these findings are vitally needed.

## Data Availability

The data are available from the corresponding author upon reasonable request.

## References

[1] Hodges H., Fealko C., Soares N. (2020). Autism spectrum disorder: Definition, epidemiology, causes, and clinical evaluation. Transl. Pediatr..

[2] Almandil N.B., Alkuroud D.N., AbdulAzeez S., AlSulaiman A., Elaissari A., Borgio J.F. (2019). Environmental and Genetic Factors in Autism Spectrum Disorders: Special Emphasis on Data from Arabian Studies. Int. J. Environ. Res. Public Health.

[3] Griffiths K.K., Levy R.J. (2017). Evidence of Mitochondrial Dysfunction in Autism: Biochemical Links, Genetic-Based Associations, and Non-Energy-Related Mechanisms. Oxid. Med. Cell. Longev..

[4] Lamanna J., Meldolesi J. (2024). Autism Spectrum Disorder: Brain Areas Involved, Neurobiological Mechanisms, Diagnoses and Therapies. Int. J. Mol. Sci..

[5] Ha S., Sohn I.J., Kim N., Sim H.J., Cheon K.A. (2015). Characteristics of Brains in Autism Spectrum Disorder: Structure, Function and Connectivity across the Lifespan. Exp. Neurobiol..

[6] Xia Q.Q., Singh A., Wang J., Xuan Z.X., Singer J.D., Powell C.M. (2024). Autism risk gene *Cul3* alters neuronal morphology via caspase-3 activity in mouse hippocampal neurons. Front. Cell. Neurosci..

[7] Kelly E., Escamilla C.O., Tsai P.T. (2021). Cerebellar Dysfunction in Autism Spectrum Disorders: Deriving Mechanistic Insights from an Internal Model Framework. Neuroscience.

[8] van der Heijden M.E., Gill J.S., Sillitoe R.V. (2021). Abnormal Cerebellar Development in Autism Spectrum Disorders. Dev. Neurosci..

[9] Mapelli L., Soda T., D’Angelo E., Prestori F. (2022). The Cerebellar Involvement in Autism Spectrum Disorders: From the Social Brain to Mouse Models. Int. J. Mol. Sci..

[10] Hu X., Li J., Fu M., Zhao X., Wang W. (2021). The JAK/STAT signaling pathway: From bench to clinic. Signal Transduct. Target. Ther..

[11] Khera R., Mehan S., Kumar S., Sethi P., Bhalla S., Prajapati A. (2022). Role of JAK-STAT and PPAR-Gamma Signalling Modulators in the Prevention of Autism and Neurological Dysfunctions. Mol. Neurobiol..

[12] Barone R., Rizzo R., Tabbì G., Malaguarnera M., Frye R.E., Bastin J. (2019). Nuclear Peroxisome Proliferator-Activated Receptors (PPARs) as Therapeutic Targets of Resveratrol for Autism Spectrum Disorder. Int. J. Mol. Sci..

[13] Wang L., Chen J., Hu Y., Liao A., Zheng W., Wang X., Lan J., Shen J., Wang S., Yang F. (2022). Progranulin improves neural development via the PI3K/Akt/GSK-3β pathway in the cerebellum of a VPA-induced rat model of ASD. Transl. Psychiatry.

[14] Jiang C.C., Lin L.S., Long S., Ke X.Y., Fukunaga K., Lu Y.M., Han F. (2022). Signalling pathways in autism spectrum disorder: Mechanisms and therapeutic implications. Signal Transduct. Target. Ther..

[15] Caracci M.O., Ávila M.E., De Ferrari G.V. (2016). Synaptic Wnt/GSK3β Signaling Hub in Autism. Neural Plast..

[16] Li Z., Zhu Y.X., Gu L.J., Cheng Y. (2021). Understanding autism spectrum disorders with animal models: Applications, insights, and perspectives. Zool. Res..

[17] Bjørklund G., Meguid N.A., El-Bana M.A., Tinkov A.A., Saad K., Dadar M., Hemimi M., Skalny A.V., Hosnedlová B., Kizek R. (2020). Oxidative Stress in Autism Spectrum Disorder. Mol. Neurobiol..

[18] Ornoy A., Weinstein-Fudim L., Ergaz Z. (2019). Prevention or Amelioration of Autism-Like Symptoms in Animal Models: Will it Bring Us Closer to Treating Human ASD?. Int. J. Mol. Sci..

[19] Liu F., Horton-Sparks K., Hull V., Li R.W., Martínez-Cerdeño V. (2018). The valproic acid rat model of autism presents with gut bacterial dysbiosis similar to that in human autism. Mol. Autism.

[20] Schiavi S., Iezzi D., Manduca A., Leone S., Melancia F., Carbone C., Petrella M., Mannaioni G., Masi A., Trezza V. (2019). Reward-Related Behavioral, Neurochemical and Electrophysiological Changes in a Rat Model of Autism Based on Prenatal Exposure to Valproic Acid. Front. Cell. Neurosci..

[21] Mehra S., Ul Ahsan A., Seth E., Chopra M. (2022). Critical Evaluation of Valproic Acid-Induced Rodent Models of Autism: Current and Future Perspectives. J. Mol. Neurosci. MN.

[22] Bjørk M.H., Zoega H., Leinonen M.K., Cohen J.M., Dreier J.W., Furu K., Gilhus N.E., Gissler M., Hálfdánarson Ó., Igland J. (2022). Association of Prenatal Exposure to Antiseizure Medication with Risk of Autism and Intellectual Disability. JAMA Neurol..

[23] Guerra M., Medici V., Weatheritt R., Corvino V., Palacios D., Geloso M.C., Farini D., Sette C. (2023). Fetal exposure to valproic acid dysregulates the expression of autism-linked genes in the developing cerebellum. Transl. Psychiatry.

[24] Aishworiya R., Valica T., Hagerman R., Restrepo B. (2022). An Update on Psychopharmacological Treatment of Autism Spectrum Disorder. Neurother. J. Am. Soc. Exp. Neurother..

[25] McKinnell Z.E., Maze T., Ramos A., Challans B., Plakke B. (2021). Valproic acid treated female Long-Evans rats are impaired on attentional set-shifting. Behav. Brain Res..

[26] Kabel A.M., Atef A., Borg H.M., El-Sheikh A.A.K., Al Khabbaz H.J., Arab H.H., Estfanous R.S. (2022). Perindopril/Ambrosin Combination Mitigates Dextran Sulfate Sodium-Induced Colitis in Mice: Crosstalk between Toll-Like Receptor 4, the Pro-Inflammatory Pathways, and SIRT1/PPAR-γ Signaling. Pharmaceuticals.

[27] Şen S., Hacıosmanoğlu E. (2022). Comparing the Neuroprotective Effects of Telmisartan, Perindopril, and Nebivolol Against Lipopolysaccharide-Induced Injury in Neuron-Like Cells. Cureus.

[28] Sayed A.M., Abdel-Fattah M.M., Arab H.H., Mohamed W.R., Hassanein E.H.M. (2022). Targeting inflammation and redox aberrations by perindopril attenuates methotrexate-induced intestinal injury in rats: Role of TLR4/NF-κB and c-Fos/c-Jun pro-inflammatory pathways and PPAR-γ/SIRT1 cytoprotective signals. Chem. Biol. Interact..

[29] Aljuhani N., Ismail R.S., El-Awady M.S., Hassan M.H. (2020). Modulatory effects of perindopril on cisplatin-induced nephrotoxicity in mice: Implication of inflammatory cytokines and caspase-3 mediated apoptosis. Acta Pharm..

[30] Banerjee A., Engineer C.T., Sauls B.L., Morales A.A., Kilgard M.P., Ploski J.E. (2014). Abnormal emotional learning in a rat model of autism exposed to valproic acid in utero. Front. Behav. Neurosci..

[31] Alzahrani Y.M., Alim ASattar M.A., Kamel F.O., Ramadan W.S., Alzahrani Y.A. (2020). Possible combined effect of perindopril and Azilsartan in an experimental model of dementia in rats. Saudi Pharm. J. SPJ Off. Publ. Saudi Pharm. Soc..

[32] Mashhoody T., Rastegar K., Zal F. (2014). Perindopril may improve the hippocampal reduced glutathione content in rats. Adv. Pharm. Bull..

[33] Elesawy R.O., El-Deeb O.S., Eltokhy A.K., Arakeep H.M., Ali D.A., Elkholy S.S., Kabel A.M. (2022). Postnatal baicalin ameliorates behavioral and neurochemical alterations in valproic acid-induced rodent model of autism: The possible implication of sirtuin-1/mitofusin-2/ Bcl-2 pathway. Biomed. Pharmacother..

[34] Zhou B., Zheng X., Chen Y., Yan X., Peng J., Liu Y., Zhang Y., Tang L., Wen M. (2022). The Changes of Amygdala Transcriptome in Autism Rat Model After Arginine Vasopressin Treatment. Front. Neurosci..

[35] Kalueff A.V., Stewart A.M., Song C., Berridge K.C., Graybiel A.M., Fentress J.C. (2016). Neurobiology of rodent self-grooming and its value for translational neuroscience. Nat. Rev. Neuroscience.

[36] Rein B., Ma K., Yan Z. (2020). A standardized social preference protocol for measuring social deficits in mouse models of autism. Nat. Protoc..

[37] Chaloob M.K., Ali H.H., Qasim B.J., Mohammed A.S. (2012). Immunohistochemical Expression of Ki-67, PCNA and CD34 in Astrocytomas: A Clinicopathological Study. Oman Med. J..

[38] Lord C., Brugha T.S., Charman T., Cusack J., Dumas G., Frazier T., Jones E.J.H., Jones R.M., Pickles A., State M.W. (2020). Autism spectrum disorder. Nat. Rev. Dis. Primers.

[39] Morris G., Puri B.K., Frye R.E., Maes M. (2018). The Putative Role of Environmental Mercury in the Pathogenesis and Pathophysiology of Autism Spectrum Disorders and Subtypes. Mol. Neurobiol..

[40] Angrand L., Masson J.D., Rubio-Casillas A., Nosten-Bertrand M., Crépeaux G. (2022). Inflammation and Autophagy: A Convergent Point between Autism Spectrum Disorder (ASD)-Related Genetic and Environmental Factors: Focus on Aluminum Adjuvants. Toxics.

[41] Mabunga D.F., Gonzales E.L., Kim J.W., Kim K.C., Shin C.Y. (2015). Exploring the Validity of Valproic Acid Animal Model of Autism. Exp. Neurobiol..

[42] Qi C., Chen A., Mao H., Hu E., Ge J., Ma G., Ren K., Xue Q., Wang W., Wu S. (2022). Excitatory and Inhibitory Synaptic Imbalance Caused by Brain-Derived Neurotrophic Factor Deficits During Development in a Valproic Acid Mouse Model of Autism. Front. Mol. Neurosci..

[43] Jiang S., Xiao L., Sun Y., He M., Gao C., Zhu C., Chang H., Ding J., Li W., Wang Y. (2022). The GABAB receptor agonist STX209 reverses the autism-like behaviour in an animal model of autism induced by prenatal exposure to valproic acid. Mol. Med. Rep..

[44] Reinhardt V.P., Iosif A.M., Libero L., Heath B., Rogers S.J., Ferrer E., Nordahl C., Ghetti S., Amaral D., Solomon M. (2020). Understanding Hippocampal Development in Young Children with Autism Spectrum Disorder. J. Am. Acad. Child Adolesc. Psychiatry.

[45] Long J., Li H., Liu Y., Liao X., Tang Z., Han K., Chen J., Zhang H. (2024). Insights into the structure and function of the hippocampus: Implications for the pathophysiology and treatment of autism spectrum disorder. Front. Psychiatry.

[46] Banker S.M., Gu X., Schiller D., Foss-Feig J.H. (2021). Hippocampal contributions to social and cognitive deficits in autism spectrum disorder. Trends Neurosci..

[47] Liu C., Liu J., Gong H., Liu T., Li X., Fan X. (2023). Implication of Hippocampal Neurogenesis in Autism Spectrum Disorder: Pathogenesis and Therapeutic Implications. Curr. Neuropharmacol..

[48] Rexrode L.E., Hartley J., Showmaker K.C., Challagundla L., Vandewege M.W., Martin B.E., Blair E., Bollavarapu R., Antonyraj R.B., Hilton K. (2024). Molecular profiling of the hippocampus of children with autism spectrum disorder. Mol. Psychiatry.

[49] Dionísio A., Espírito A., Pereira A.C., Mouga S., d’Almeida O.C., Oliveira G., Castelo-Branco M. (2024). Neurochemical differences in core regions of the autistic brain: A multivoxel 1H-MRS study in children. Sci. Rep..

[50] Elibol B., Kilic U. (2018). High Levels of SIRT1 Expression as a Protective Mechanism Against Disease-Related Conditions. Front. Endocrinol..

[51] Bu X., Wu D., Lu X., Yang L., Xu X., Wang J., Tang J. (2017). Role of SIRT1/PGC-1α in mitochondrial oxidative stress in autistic spectrum disorder. Neuropsychiatr. Dis. Treat..

[52] Salminen A., Kaarniranta K., Kauppinen A. (2013). Crosstalk between Oxidative Stress and SIRT1: Impact on the Aging Process. Int. J. Mol. Sci..

[53] Liu F.J., Gu T.J., Wei D.Y. (2022). Emodin alleviates sepsis-mediated lung injury via inhibition and reduction of NF-kB and HMGB1 pathways mediated by SIRT1. Kaohsiung J. Med. Sci..

[54] Zhu Z., Li H., Chen W., Cui Y., Huang A., Qi X. (2020). Perindopril Improves Cardiac Function by Enhancing the Expression of SIRT3 and PGC-1α in a Rat Model of Isoproterenol-Induced Cardiomyopathy. Front. Pharmacol..

[55] Al-Harbi N.O., Nadeem A., Ahmad S.F., Al-Ayadhi L.Y., Al-Harbi M.M., As Sobeai H.M., Ibrahim K.E., Bakheet S.A. (2020). Elevated expression of toll-like receptor 4 is associated with NADPH oxidase-induced oxidative stress in B cells of children with autism. Int. Immunopharmacol..

[56] Szabó D., Tod P., Gölöncsér F., Román V., Lendvai B., Otrokocsi L., Sperlágh B. (2022). Maternal P2X7 receptor inhibition prevents autism-like phenotype in male mouse offspring through the NLRP3-IL-1β pathway. Brain Behav. Immun..

[57] Malaguarnera M., Khan H., Cauli O. (2020). Resveratrol in Autism Spectrum Disorders: Behavioral and Molecular Effects. Antioxidants.

[58] Shen Y., Qian L., Luo H., Li X., Ruan Y., Fan R., Si Z., Chen Y., Li L., Liu Y. (2022). The Significance of NLRP Inflammasome in Neuropsychiatric Disorders. Brain Sci..

[59] Wong R.S.Y. (2022). Neuroinflammation in autism spectrum disorders: Potential target for mesenchymal stem cell-based therapy. Egypt. J. Neurol. Psychiatry Neurosurg..

[60] Ahmad S.F., Ansari M.A., Nadeem A., Alzahrani M.Z., Bakheet S.A., Attia S.M. (2018). Resveratrol Improves Neuroimmune Dysregulation Through the Inhibition of Neuronal Toll-Like Receptors and COX-2 Signaling in BTBR T+ Itpr3tf/J Mice. Neuromol. Med..

[61] El-Shoura E.A.M., Messiha B.A.S., Sharkawi S.M.Z., Hemeida R.A.M. (2018). Perindopril ameliorates lipopolysaccharide-induced brain injury through modulation of angiotensin-II/angiotensin-1-7 and related signaling pathways. Eur. J. Pharmacol..

[62] Zhang X., Zhao D., Feng J., Yang X., Lan Z., Yang T., Kong X., Qu H., Zhou H. (2021). LuQi Formula Regulates NLRP3 Inflammasome to Relieve Myocardial-Infarction-Induced Cardiac Remodeling in Mice. Evid. Based Complement. Altern. Med. ECAM.

[63] Valdivia A.O., Farr V., Bhattacharya S.K. (2019). A novel myelin basic protein transcript variant in the murine central nervous system. Mol. Biol. Rep..

[64] Martinsen V., Kursula P. (2022). Multiple sclerosis and myelin basic protein: Insights into protein disorder and disease. Amino Acids.

[65] Graciarena M., Seiffe A., Nait-Oumesmar B., Depino A.M. (2019). Hypomyelination and Oligodendroglial Alterations in a Mouse Model of Autism Spectrum Disorder. Front. Cell. Neurosci..

[66] Gonzalez-Gronow M., Cuchacovich M., Francos R., Cuchacovich S., Blanco A., Sandoval R., Gomez C.F., Valenzuela J.A., Ray R., Pizzo S.V. (2015). Catalytic autoantibodies against myelin basic protein (MBP) isolated from serum of autistic children impair in vitro models of synaptic plasticity in rat hippocampus. J. Neuroimmunol..

[67] Jain M., Singh M.K., Shyam H., Mishra A., Kumar S., Kumar A., Kushwaha J. (2021). Role of JAK/STAT in the Neuroinflammation and its Association with Neurological Disorders. Ann. Neurosci..

[68] Patel A.B., Tsilioni I., Leeman S.E., Theoharides T.C. (2016). Neurotensin stimulates sortilin and mTOR in human microglia inhibitable by methoxyluteolin, a potential therapeutic target for autism. Proc. Natl. Acad. Sci. USA.

[69] Tufano M., Pinna G. (2020). Is There a Future for PPARs in the Treatment of Neuropsychiatric Disorders?. Molecules.

[70] Panzer U., Zahner G., Wienberg U., Steinmetz O.M., Peters A., Turner J.E., Paust H.J., Wolf G., Stahl R.A., Schneider A. (2008). 15-deoxy-Delta12,14-prostaglandin J2 inhibits INF-gamma-induced JAK/STAT1 signalling pathway activation and IP-10/CXCL10 expression in mesangial cells. Nephrol. Dial. Transplant. Off. Publ. Eur. Dial. Transpl. Assoc. Eur. Ren. Assoc..

[71] El-Shoura E.A.M., Sharkawi S.M.Z., Messiha B.A.S., Bakr A.G., Hemeida R.A.M. (2019). Perindopril mitigates LPS-induced cardiopulmonary oxidative and inflammatory damage via inhibition of renin angiotensin system, inflammation and oxidative stress. Immunopharmacol. Immunotoxicol..

[72] Zhang J., Zhang J.X., Zhang Q.L. (2016). PI3K/AKT/mTOR-mediated autophagy in the development of autism spectrum disorder. Brain Res. Bull..

[73] Choi C.S., Gonzales E.L., Kim K.C., Yang S.M., Kim J.W., Mabunga D.F., Cheong J.H., Han S.H., Bahn G.H., Shin C.Y. (2016). The transgenerational inheritance of autism-like phenotypes in mice exposed to valproic acid during pregnancy. Sci. Rep..

[74] Zakaria S., Allam S., El-Sisi A.E. (2022). Perindopril sensitizes hepatocellular carcinoma to chemotherapy: A possible role of leptin/Wnt/β-catenin axis with subsequent inhibition of liver cancer stem cells. Saudi Pharm. J. SPJ Off. Publ. Saudi Pharm. Soc..

[75] Jin Y., Choi J., Lee S., Kim J.W., Hong Y. (2019). Pathogenetical and Neurophysiological Features of Patients with Autism Spectrum Disorder: Phenomena and Diagnoses. J. Clin. Med..

[76] Zhao P., Fu H., Cheng H., Zheng R., Yuan D., Yang J., Li S., Li E., Li L. (2022). Acupuncture at ST36 Alleviates the Behavioral Disorder of Autistic Rats by Inhibiting TXNIP-Mediated Activation of NLRP3. J. Neuropathol. Exp. Neurol..

